# Pharmacological inhibition of the RhoA pathway by melatonin reduces viral replication and proinflammatory response against ZIKV and DENV-4 neuroinfections

**DOI:** 10.3389/fimmu.2025.1630116

**Published:** 2025-08-01

**Authors:** José De Jesus Bravo-Silva, Ricardo Jimenez-Camacho, Magda Lizbeth Benítez-Vega, Jonathan Hernández-Castillo, Carlos Daniel Cordero-Rivera, Carlos Noe Farfan-Morales, Marcos Pérez-García, Raymundo Cruz, Rosa María del Ángel

**Affiliations:** Department of Infectomics and Molecular Pathogenesis, Center for Research and Advanced Studies (CINVESTAV), Mexico City, Mexico

**Keywords:** inflammation, melatonin, brain, cerebellum, viral infection, ZIKV, DENV-4

## Abstract

**Introduction:**

Zika virus (ZIKV) and dengue virus (DENV) are mosquito-borne flaviviruses associated with serious neurological complications, such as Guillain-Barré syndrome and congenital Zika syndrome (ZIKV), as well as encephalitis, meningitis, and stroke (DENV). Despite their growing public health impact in tropical regions, there are currently no specific treatments available. Both viruses modulate the RhoA GTPase pathway, which is involved in immune regulation and cytoskeletal dynamics. Melatonin, a hormone with antioxidant and immunomodulatory properties, has previously been shown to inhibit Japanese encephalitis virus (JEV) replication through RhoA pathway modulation.

**Methods:**

We evaluated the antiviral potential of melatonin against ZIKV and DENV-4 in vitro using U87-MG cells and in vivo using two mouse models: immunodeficient AG129 and neonatal immunocompetent CD1 mice. Antiviral and immunomodulatory effects were assessed by quantitative RT-PCR and analysis of inflammatory markers, including interferon-stimulated genes (MX1, IFI44L, IFN-β) and cytokines (IL-1β, TNF-α). Microglial activation and polarization were also analyzed in brain tissues.

**Results:**

Melatonin treatment significantly reduced ZIKV and DENV-4 replication and the associated inflammatory response in U87-MG cells. In AG129 mice, melatonin increased survival, attenuated clinical signs during DENV-4 infection, and reduced viral genome copies of both viruses. In neonatal CD1 mice, melatonin markedly decreased viral loads in the brain and suppressed inflammatory gene expression, microglial activation, and M1/M2 polarization imbalance.

**Discussion:**

Our findings demonstrate that melatonin exerts both antiviral and anti-inflammatory effects against ZIKV and DENV-4 infections in vitro and in vivo, likely through inhibition of the RhoA signaling pathway. These results suggest that melatonin is a promising therapeutic candidate for neuroinfections caused by flaviviruses.

## 1 Introduction

Zika virus (ZIKV) and dengue virus (DENV) are arthropod-borne pathogens belonging to the *Flaviviridae* family. Both viruses currently lack specific treatments, becoming a significant public health challenge in tropical regions. ZIKV infection has been associated with Guillain-Barré syndrome in adults and congenital Zika syndrome in newborns. Severe dengue cases can result in complications such as encephalitis, viral meningitis, and stroke—conditions that remain poorly studied and may lead to long-term cognitive impairment. Current estimates indicate that ZIKV and DENV infections contribute substantially to global morbidity and mortality, with particular concern over congenital disabilities resulting from maternal ZIKV infection. Although studies on DENV as a neurotropic virus remain limited, DENV-4 infection of neurons, endothelial cells, and microglia has been associated with functional and structural alterations in susceptible mice, resembling the molecular mechanisms described for ZIKV (1, 2). Addressing these emerging threats requires a deeper understanding of the complex molecular mechanisms underlying viral pathogenesis, which is essential for developing effective therapeutic strategies (3).

Melatonin, a neurohormone primarily secreted by the pineal gland, has garnered considerable attention due to its role in regulating circadian rhythms, as well as its immunomodulatory and neuroprotective properties (4). Research has suggested that melatonin can modulate immune responses and influence multiple signaling pathways within the central nervous system, including the RhoA signaling pathway (4, 5). RhoA GTPase activity is critical for various cellular processes such as cytoskeletal dynamics, cell migration, immune regulation, and apoptosis (6, 7). Recent studies have implicated the RhoA pathway in the pathogenesis of several viral infections, including Japanese Encephalitis virus (JEV), Ebola virus (EBOV), and Severe Acute Respiratory Syndrome Coronavirus 2 (SARS-CoV-2) (8–10).

Interestingly, the evidence indicates that modulating RhoA activity by melatonin can improve host cell resilience, enhancing immune responses against viral infections (9–11). These findings highlight a promising therapeutic approach for combating neuroinfections caused by *Flaviviridae* viruses. However, the complex interaction between melatonin signaling, RhoA pathway activation, and antiviral responses remains largely unexplored.

Our results suggest that the pharmacological modulation of the RhoA pathway by melatonin could represent an innovative therapeutic strategy to enhance antiviral and immunomodulatory responses against neurotropic viruses such as ZIKV and DENV-4.

## 2 Materials and methods

### 2.1 Cell culture and reagents

U87-MG is glioblastoma cells, kindly provided by Dr. Olivia Hernández-González (Instituto Nacional de Rehabilitación [INR], Mexico), were cultured in Advanced DMEM (GIBCO, 12491015) supplemented with 13% fetal bovine serum (FBS, GIBCO, 12657-02), 1 U/mL penicillin/streptomycin (Sigma, P4333), and 2.5 mM glutamine (Sigma-Aldrich, G8540). Experiments with U87-MG cells were performed in three independent replicates.

### 2.2 Animal models

All experimental animal procedures followed the Mexican Official Standard for the Production, Care, and Use of Laboratory Animals (NOM-062-ZOO-1999). The protocols for the use of AG129 mice (129/Sv strain; 6–8 weeks old, both male and female), obtained from Marshall Bioresources, UK (protocol 048-02), and neonatal CD1 mice (5–7 days old) (ICR-CD1, strain code: 022) (protocol 0382-24) were approved by the Institutional Animal Care and Use Committee (IACUC) of CINVESTAV-IPN.

AG129 and CD1 mice were housed under controlled conditions in the Animal Production and Experimentation Unit (UPEAL-CINVESTAV) with ad libitum access to food and water. Euthanasia of adult mice and neonates was performed in a 10-liter CO_2_ chamber, using a displacement rate of 30% to 70% of the chamber volume per minute, in accordance with the euthanasia of rodents using carbon dioxide following AVMA (American Veterinary Medical Association) guidelines. For adult mice, CO_2_ was administered for 5 minutes; for neonates, the exposure time was extended to 10 minutes due to their greater resistance to hypoxia. In both cases, a physical method of decapitation was applied after CO_2_ exposure to ensure death. The entire procedure was conducted under veterinary supervision and in compliance with institutional protocols and current ethical regulations.

### 2.3 Viral infection

Zika virus (ZIKV, MEX_CIENI551 strain) was kindly provided by Dr. Jesús Torres (Escuela Nacional de Ciencias Biológicas [ENCB], Instituto Politécnico Nacional, Mexico), and dengue virus serotype 4 (DENV-4, H241 strain) was donated by the Dr. Manuel Martínez Báez Institute of Epidemiological Diagnosis and Reference (InDRE, Mexico).

Both viruses were propagated in the brains of neonatal CD1 mice at the UPEAL-CINVESTAV facility. Lysates from infected and uninfected tissues were prepared and used to infect U87-MG cells at a multiplicity of infection (MOI) of 5. *In vivo* experiments were conducted by inoculating AG129 and CD1 mice with 1 × 10^6^ focus-forming units (FFU) of ZIKV or 1 × 10^7^ FFU of DENV-4. Negative control groups were administered an equivalent volume of saline solution under the same experimental conditions.

### 2.4 Drugs and reagents

Cells were treated with melatonin (Sigma-Aldrich, M5250), which was dissolved in 40% ethanol at a concentration of 125 mM and stored in aliquots at −20°C until use. Final concentrations of 0.6 mM and 1.2 mM were initially tested *in vitro*; subsequently, the 1.2 mM dose was selected for all further experiments. To inhibit the RhoA pathway, Rho Inhibitor I (Cytoskeleton, *Clostridium difficile* toxin B [CT04]) was reconstituted according to the manufacturer’s instructions, aliquoted, and stored at −20°C. The final working concentration was 1.0 μg/mL. In *in vivo* experiments, liquid melatonin (Vitamatic, USA) was administered at 20 mg/kg to AG129 mice and 10 mg/kg to neonatal CD1 mice, according to body weight, without altering the original formulation.

### 2.5 Cell viability assessment

Cell viability in the presence of melatonin (MEL) was evaluated in U87-MG cells using the colorimetric 3-(4,5-dimethylthiazol-2-yl)-2,5-diphenyltetrazolium bromide (MTT) assay (Sigma-Aldrich). Cells were seeded in flat-bottom 96-well plates and treated with various concentrations of MEL (0.15, 0.3, 0.6, and 1.2 mM) for 24 and 48 hours. Following treatment, MTT reagent was added to each well, and plates were incubated at 37°C in a 5% CO_2_ atmosphere for the time specified by the manufacturer’s instructions. Formazan crystals were then solubilized with DMSO, and absorbance was measured at 562 nm, with 630 nm as the reference wavelength to correct for background absorbance.

Vehicle-treated cells (40% ethanol) were used as controls and were considered to represent 100% viability. Across all tested concentrations, cell viability remained above 80% after both 24 and 48 hours of treatment.

### 2.6 Confocal microscopy

U87-MG cells were cultured on coverslips in 24-well plates until reaching 70–80% confluence. Cells were infected or mock-infected with ZIKV or DENV-4 by exposure to the virus for 2 hours. Following infection, cells were washed, replaced with fresh medium and treated with MEL (0.6 or 1.2 mM) for 48 hours post-infection (hpi).

For experiments involving the RhoA inhibitor CT04, U87-MG cells were cultured under the same conditions, infected as described, and then incubated with serum-free DMEM supplemented with 1.0 μg/mL CT04 for 48 hpi.

At the end of the treatments, cells were washed three times with 1× PBS, fixed with 4% paraformaldehyde (PFA) for 30 minutes at 4 °C, and permeabilized with 0.2% saponin, 1% fetal bovine serum (FBS), and 1× PBS for 30 minutes at room temperature. Cells were then incubated overnight at 4 °C with primary antibodies specific for viral infection detection: ZIKV anti-NS3 protein (1:300, Genetex, GTX133309), DENV anti-NS3 protein (1:300, Genetex, GTX124252) and anti-IBA1 (1:200, Santa Cruz, sc-32725), Alexa Fluor 555-conjugated anti-rabbit secondary antibody (1:300, Life Technologies) and Alexa Fluor 488-conjugated anti-rabbit secondary antibody (1:750, Life Technologies), were used to detect the primary antibodies, and nuclei were counterstained with Hoechst (1:1000, Santa Cruz Biotechnology). Images were acquired using a Leica TCS SP8 confocal microscope (Leica Microsystems) and processed with Leica Application Suite X Core Offline v3.3.0 software.

### 2.7 Immunofluorescent staining of frozen tissue

For brain and cerebellum sections of AG129 and CD1 mice, frozen tissue from the left lobe of three mice per condition was processed according to the protocol described by Trist, Wright, and Rangel (12). The following antibodies were used to detect viral infection and cellular markers of inflammation: ZIKV anti-NS3 protein (1:300, Genetex, GTX133309), DENV anti-NS3 protein (1:300, Genetex, GTX124252), anti-Iba1 (1:200, Santa Cruz, sc-32725), anti-CD86 (1:150, Cell Signaling, 19589) and anti-CD206 (1:150, Cell Signaling, 24595). An Alexa Fluor 555-conjugated anti-rabbit secondary antibody (1:300, Life Technologies) and Alexa Fluor 488-conjugated anti-rabbit secondary antibody (1:750, Life Technologies), were used to detect the primary antibodies, and nuclei were counterstained with Hoechst (1:1000, Santa Cruz Biotechnology).

Samples were analyzed using the Leica SP8 confocal microscope. Image processing was performed using LASX Office 1.4.7–28921 software. Three fields from three independent experiments were analyzed.

### 2.8 Focus-forming unit assay

A focus-forming unit (FFU) assay was performed to quantify the number of infectious viral particles produced in MEL-treated cells. Supernatants from infected and mock-infected cells were processed according to. The FFU assays were performed to quantify infectious ZIKV and DENV-4 particles. Vero cells were seeded in 96-well plates and infected with serial dilutions of viral supernatants for 2 h at 37°C. After infection, cells were overlaid with a medium DMEM and incubated for 48 h. Cells were then fixed with 4% paraformaldehyde and permeabilized with 0.1% Triton X-100. Viral foci were detected by staining with the 4G2 antibody (anti- Envelope 1:300, Genetex, GTX57154) and analyzed by epifluorescence microscopy using a Leica DM IL microscope.

### 2.9 Quantitative real-time PCR

*In vitro* assays were performed to quantify viral genome copy number and evaluate the expression of proinflammatory and interferon-stimulated genes (ISGs) from cell monolayers. Total RNA was extracted using TRIzol Reagent (Invitrogen, Cat. 15596026.PPS). For *in vivo* analysis, from 50 mg of tissue collected from the right cerebral lobe, including the cerebellum, of three mice per condition (each mouse considered an individual experimental unit). These samples were used to determine viral load and assess the expression of immune response-related genes. According to the manufacturer’s instructions. Genomic DNA contamination was eliminated by treatment with DNase I (New England Biolabs, Cat. M0303).

Quantitative real-time PCR (qPCR) was performed to quantify ZIKV and DENV-4 RNA copies in brain and cerebellar tissues. Reactions were carried out in a final volume of 10 μL containing 5 μL of qPCRBIO SyGreen BlueMix Lo-ROX (PCR Biosystems, PB20.15), 0.4 μL of each primer (10 μM) (**Table 1**), and 1 μL of diluted cDNA, with RNase-free water added to adjust the volume.

**Table 1:** Mouse and human primers were used for qPCR analysis.

Primer	Sequence 5^”^- 3^”^
*F-TNFa mouse*	GAA CTG GCA GAA GAG GCA CT
*R-TNFa mouse*	AGG GTC TGG GCC ATA GAA CT
*F-IL-1β mouse*	CTC ATT GTG GCT GTG GAG AA
*R-IL-1β mouse*	TCT AAT GGG AAC GTC ACA CA
*F-NF-κB mouse*	CTGGTGGACACATACAGGAAGAC
*R-NF-κB mouse*	ATAGGCACTGTCTTCTTTCACCTC
*F-IL1β Human*	GTGGCATTCAAGGAGTACCTC
*R-IL1β Human*	TGATGGCCTTCGATTCTGGATT
*F-TNFα Human*	CCTCTCTCTAATCAGCCCTCTG
*R-TNFα Human*	GAGGACCTGGGAGTAGATGAG
*F-IFN-β Human*	GATGAACTTTGACATCCCTGAG
*R-IFN-β Human*	TAGCAAAGATGTTCTGGAGCA
*F-MX1 Human*	GCATCCCACCCTCTATTACT
*R-MX1 Human*	ACCTTCTCCTCATACTGGCTG
*F-IFI44L Human*	GATTGCAGTGAGGTTCTTC
*R-IFI44L Human*	GATGAACTTTGACATCCCTGAG

This table lists the forward and reverse primer sequences used for gene expression analysis by quantitative PCR (qPCR) in mouse and human samples.

Thermocycling conditions were: 50°C for 10 min, 95°C for 2 min, followed by 40 cycles of 95°C for 30 s and 60°C for 30 s.

The assay was performed on an Eco Real-Time PCR System (Illumina), and quantification was achieved using a standard curve generated from serial dilutions (10^10^ to 10² copies) of a 220-bp fragment of the ZIKV and DENV-4 genomes, amplified using general Orthoflavivirus primers as described by Maher-Sturgess et al. (2008). The detection threshold was established using RNA from an uninfected control mouse. Data were analyzed with EcoStudy software (v5.048909) and expressed as viral copies/50 mg of tissue.

### 2.10 RhoA activity assay

RhoA pathway activation was assessed using the RhoA Activation Assay Kit (Cytoskeleton, Denver, CO) according to the manufacturer’s instructions.

U87-MG cells were cultured in 24-well plates until reaching 80% confluence. Cells were then infected with ZIKV or DENV-4 at a multiplicity of infection (MOI) of 5 and treated with 1.2 mM MEL for 18, 24, or 48 hpi. Control groups included uninfected cells and cells treated with HBSS medium alone. Three independent experiments were conducted for each condition.

At the indicated time points, cells were lysed and immediately frozen in liquid nitrogen, then stored at −80°C until further processing.

### 2.11 Survival assays in immunodeficient AG129 mice

AG129 mice are widely used as a canonical model for ZIKV and DENV infections, primarily due to their high susceptibility to flavivirus infection and their capacity to exhibit consistent and observable clinical signs of disease. The mice were randomly selected for each experimental group, separated only by sex. AG129 mice were infected intraperitoneally with 4×10^6^ PFU/mL of ZIKV or 4×10^7^ PFU/mL of DENV-4, delivering 100 μL of inoculum per mouse. Control mice were inoculated with virus-free tissue lysates.

MEL treatment started four days post-infection (dpi). Mice in the treatment groups received 20 mg/kg of MEL daily via oral gavage, while infected control mice received the same volume of water. Methodology was adapted and modified from Cecon et al. (8). Body weight and clinical signs of disease were recorded daily until euthanasia. Disease severity was scored using a clinical scoring system described by Orozco et al. (13), where 1 indicates healthy mice and 5 represents moribund animals (**Supplementary Figure 1**).

Three independent experiments were performed, each including one or two mice per group. Animals were excluded from the analysis if death occurred before the expected onset of clinical signs associated with viral infection (i.e., before day 3 post infection), in the absence of weight loss or neurological symptoms, and without detectable viral RNA in serum or brain tissue (assessed by qRT-PCR). These criteria allowed us to distinguish deaths likely due to procedural problems (e.g., complications related to gastric tube or stress) from those attributable to the infection Specifically, animals that reached a cumulative clinical score of 5—based on weight loss, neurological signs, and general condition—were humanely euthanized to prevent unnecessary suffering. Euthanasia was performed using a CO_2_ chamber.

Survival data, clinical scores, and body weight were graphically represented and analyzed using GraphPad Prism 10 software.

### 2.12 Neonatal immunocompetent CD1

CD1 mice aged 5–7 days were infected intraperitoneally with 1×10^7^ PFU/mL of ZIKV or DENV-4, diluted in physiological saline to a final volume of 20 μL per mouse.

The experimental design included a time-course analysis, with day 0 corresponding to the day of inoculation. MEL treatment (10 mg/kg) was administered daily by oral gavage from day 1 to day 7 post-infection (dpi), following the protocol described by Cecon et al. and Francis et al. (8, 14). Mice were euthanized and collected at days 3 (n=9), 5 (n=9), and 7 (n=9) dpi.

Two independent experiments were performed using litters of three mice per virus at each time point. Viral load and the expression of immune response-related genes were assessed by qPCR as described above. Additionally, frozen brain sections were processed and immunolabeled for analysis by confocal microscopy. Data were graphically represented and analyzed using GraphPad Prism 10 software.

### 2.13 Quantification and statistical analysis

Confocal microscopy images were analyzed using LASX Office software (v1.4.7 28921). One-way ANOVA followed by Mann–Whitney test was used to compare group means in the FFU assays, qPCR results, RhoA activity assays, and mean fluorescence intensity (MFI) measurements.

For *in vivo* assays, Kaplan-Meier survival curves were generated, and survival rates between treated and untreated groups were compared using the Wilcoxon and Mantel-Cox tests. The ANOVA-LSD test was also used to compare mean survival times between groups. A p-value ≤ 0.05 was considered statistically significant in all analyses.

## 3 Results

### 3.1 RhoA inhibition reduces ZIKV and DENV-4 replication in U87-MG cells

**Figure 1:**
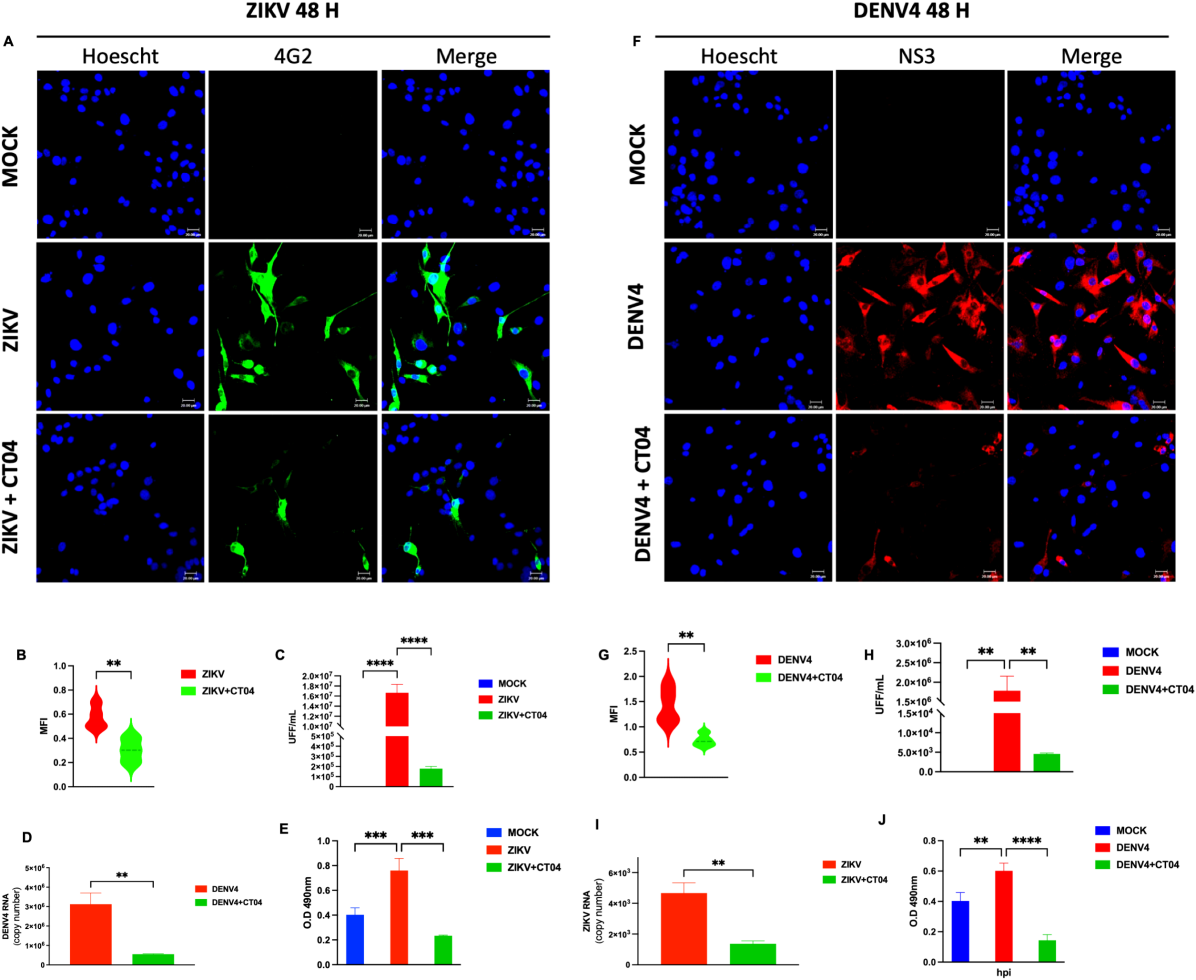
Inhibition of RhoA with Clostridium difficile toxin B (CT04) suppresses ZIKV and DENV-4 replication in U87-MG cells. U87-MG glioblastoma cells were infected with ZIKV or DENV-4 and treated with 1.0 µg/mL of CT04 for 48 hpi. RhoA inhibition led to a marked reduction in viral protein expression **(A, F)**, the mean fluorescence intensity **(B, G)**. Nuclei were counterstained with Hoechst (blue), compared to untreated infected controls. This effect correlated with a significant decrease in infectious viral particles, measured by focus-forming unit (FFU) assay **(C, H)**, and a reduction in viral genome copies as quantified by qPCR **(D, I)**. ELISA-based quantification of RhoA activation **(E, J)**. Bar graphs represent mean values ± SD from three independent replicates. Asterisks indicate statistical significance of Mann–Whitney test: ns = not significant, *p ≤ 0.05, **p ≤ 0.01, ***p ≤ 0.001, ****p ≤ 0.0001.

The RhoA signaling pathway is critical for neurodevelopment and regulates various cellular processes, including cytoskeletal remodeling and immune responses. Previous studies have demonstrated that several viruses, including EBOV, respiratory syncytial virus (RSV), rotavirus (RV), porcine pseudorabies virus (PRV), and SARS-CoV-2, hijack RhoA pathway to facilitate infection (10, 15–17). The concentration of 1 μg/mL was recommended by the manufacturer for use in neural-derived cells such as U87-MG. Additionally, a cytotoxicity analysis using the MTT assay confirmed that this concentration is safe for use in our cell cultures (**Supplementary Figure 2**). In many cases, RhoA activation is proviral, whereas its inhibition leads to antiviral effects. Our results demonstrate a direct relationship between RhoA activation and the replication of ZIKV and DENV-4. Immunofluorescence microscopy revealed that a dose of 1 μg/mL led to a reduction in viral protein expression (ZIKV, green; DENV-4, red) upon RhoA inhibition (**Figures 1A, F**), accompanied by decreased mean fluorescence intensity (MFI) (**Figures 1B, G**). Reductions in infectious viral particles, as measured by focus-forming units (FFU/mL) (**Figures 1C, H**), and viral genome copy numbers by qPCR (**Figures 1D, I**) further supported these findings. Furthermore, a reduction in the active form of RhoA was confirmed at 48 hpi following treatment with 1 µg/mL of CT04 (**Figures 1E, J**).

These results suggest that CT04, a RhoA pathway inhibitor, interferes with the replication of ZIKV and DENV-4.

### 3.2 Antiviral activity of melatonin against ZIKV and DENV-4 in U87-MG cells

MEL is a hormone produced by the pineal gland, widely used for managing sleep disorders, anxiety, and seasonal depression (4, 18). Recent studies have revealed its antioxidant, anti-inflammatory, and immunomodulatory properties (8, 9), suggesting potential antiviral effects. To explore its antiviral potential, U87-MG cells were treated with increasing concentrations of MEL (0.15, 0.3, 0.6, and 1.2 mM) and subjected to cytotoxicity assays (MTT) at 24 and 48 hours post-treatment. In all conditions, cell viability remained above 80% (**Supplementary Figure 3**), indicating that the tested concentrations were safe for further analysis. Based on these results, 0.6 and 1.2 mM were selected for subsequent experiments.

**Figure 2:**
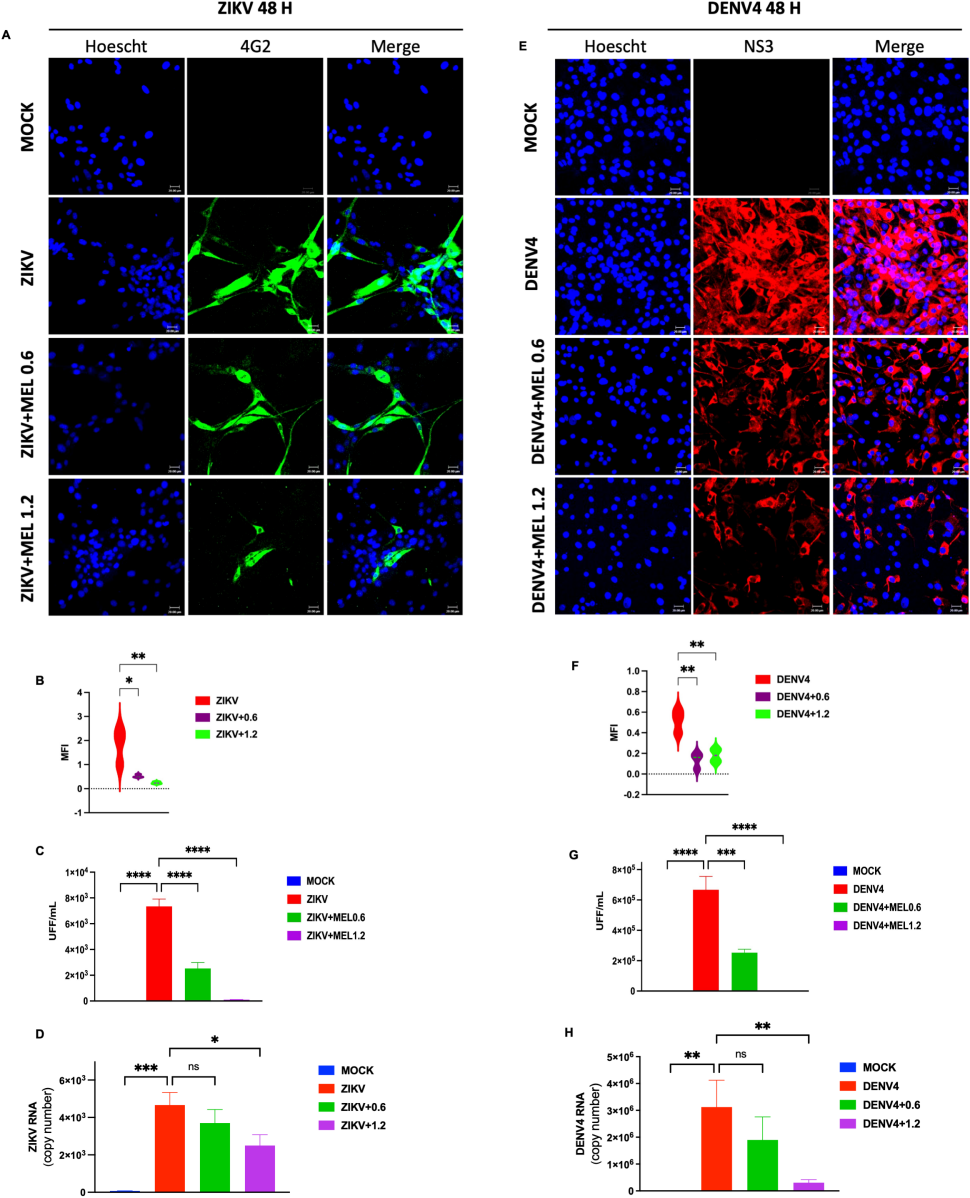
Melatonin 0.6 and 1.2 mM reduces ZIKV and DENV-4 infection in U87-MG cells dose-dependent at 48 hpi. U87-MG glioblastoma cells were infected with ZIKV or DENV-4 and treated with 0.6 and 1.2 mM for 48 hpi. The marked reduction in viral protein expression **(A, E)**, the mean fluorescence intensity **(B, F)**. Nuclei were counterstained with Hoechst (blue), compared to untreated infected controls. This effect correlated with a significant decrease in infectious viral particles, measured by focus-forming unit (FFU) assay **(C, G)**, and a reduction in viral genome cpies as quantified by qPCR **(D, H)**. Bar graphs represent mean values ± SD from three independent replicates. Asterisks indicate statistical significance of Mann–Whitney test: ns = not significant, *p ≤ 0.05, **p ≤ 0.01, ***p ≤ 0.001, ****p ≤ 0.0001.

U87-MG cells infected with ZIKV or DENV-4 (MOI 5) and treated with 0.6 or 1.2 mM MEL, showed a marked reduction in viral protein expression by confocal microscopy (**Figures 2A–E**), as evidenced by decreased MFI (**Figures 2B, F**). FFU assays (**Figures 2C, G**) and qPCR analyses (**Figures 2D, H**) confirmed significant reductions in viral titers and genome copy numbers, respectively. These findings indicate that MEL inhibits ZIKV and DENV-4 replication in U87-MG cells, with a stronger effect at 1.2 mM.

### 3.3 Melatonin modulates the inflammatory response induced by ZIKV and DENV-4

**Figure 3:**
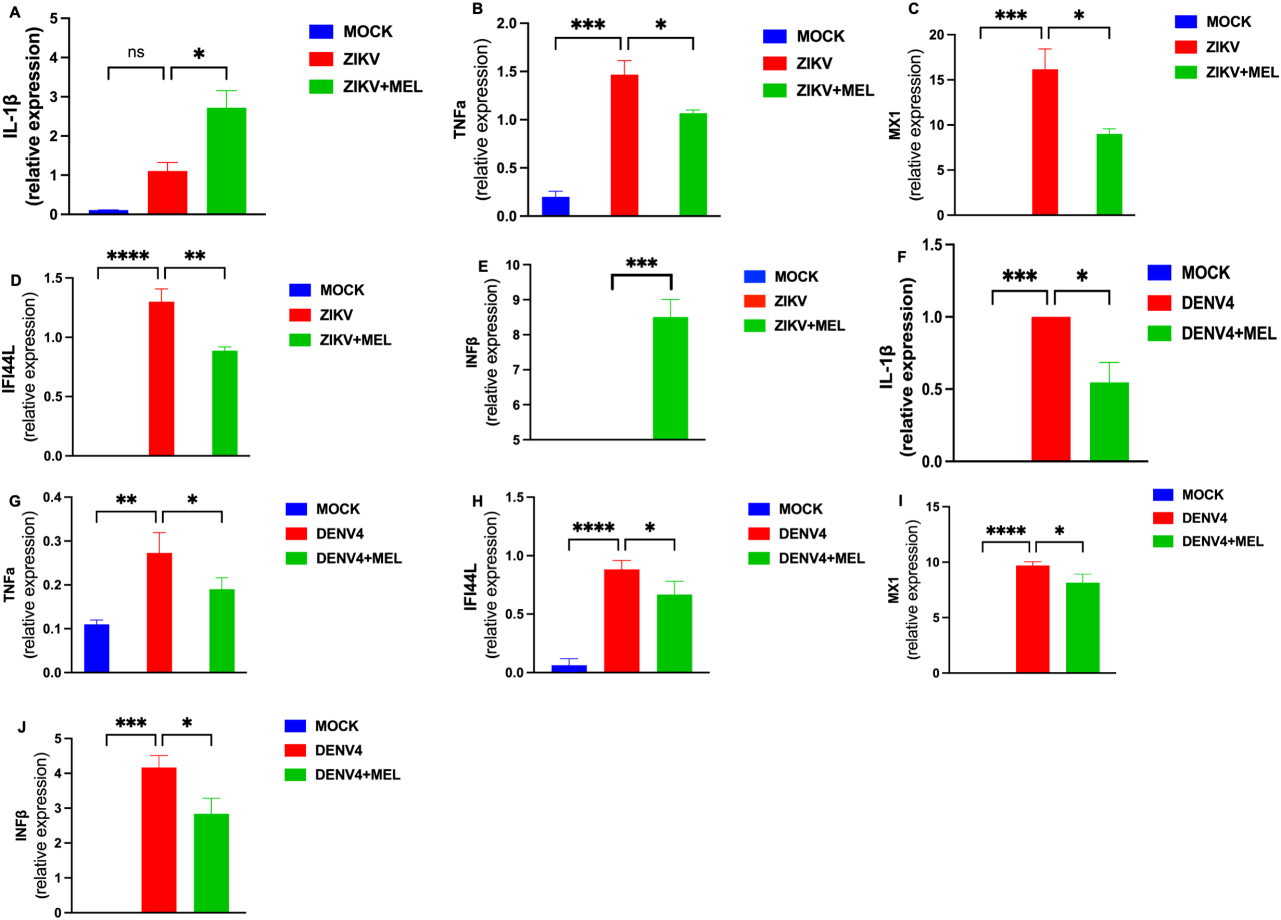
Melatonin 1.2 mM treatment reduces the inflammatory response induced by ZIKV and DENV-4 in U87-MG cells. U87-MG cells were infected for 2 hours and treated with 1.2 mM melatonin for 48 hpi. The expression of inflammatory genes and interferon-stimulated genes (ISGs) was evaluated by qPCR and normalized to GAPDH during ZIKV **(A–E)** and DENV-4 **(F–J)** infections. Melatonin treatment effectively modulated the differential immune responses induced by both viruses. Bar graphs represent mean values ± SEM from representative experiments. Bar graphs represent mean values ± SD from three independent replicates. Asterisks indicate statistical significance of Mann–Whitney test: ns = not significant, *p ≤ 0.05, **p ≤ 0.01, ***p ≤ 0.001, ****p ≤ 0.0001.

Given the role of ZIKV and DENV-4 in glial infection and neuroinflammation, we assessed the impact of MEL on inflammatory gene expression in infected U87-MG cells. Treatment with 1.2 mM MEL significantly reduced the expression of key pro-inflammatory genes (IL-1β and TNFα) and ISGs (MX1, IFI44L, and IFNβ) at 48 hpi. During ZIKV infection (**Figures 3A–E**), MEL reduces expression of TNFα, MX1 and IFI44L but significantly increases expression of IL-1β and IFNβ. In contrast, during DENV-4 infection (**Figures 3F–J**), MEL consistently reduced the expression of all evaluated genes. These results suggest that melatonin exerts virus-specific immunomodulatory effects during Orthoflavivirus infections.

### 3.4 Melatonin inhibits RhoA pathway activation and viral replication

**Figure 4:**
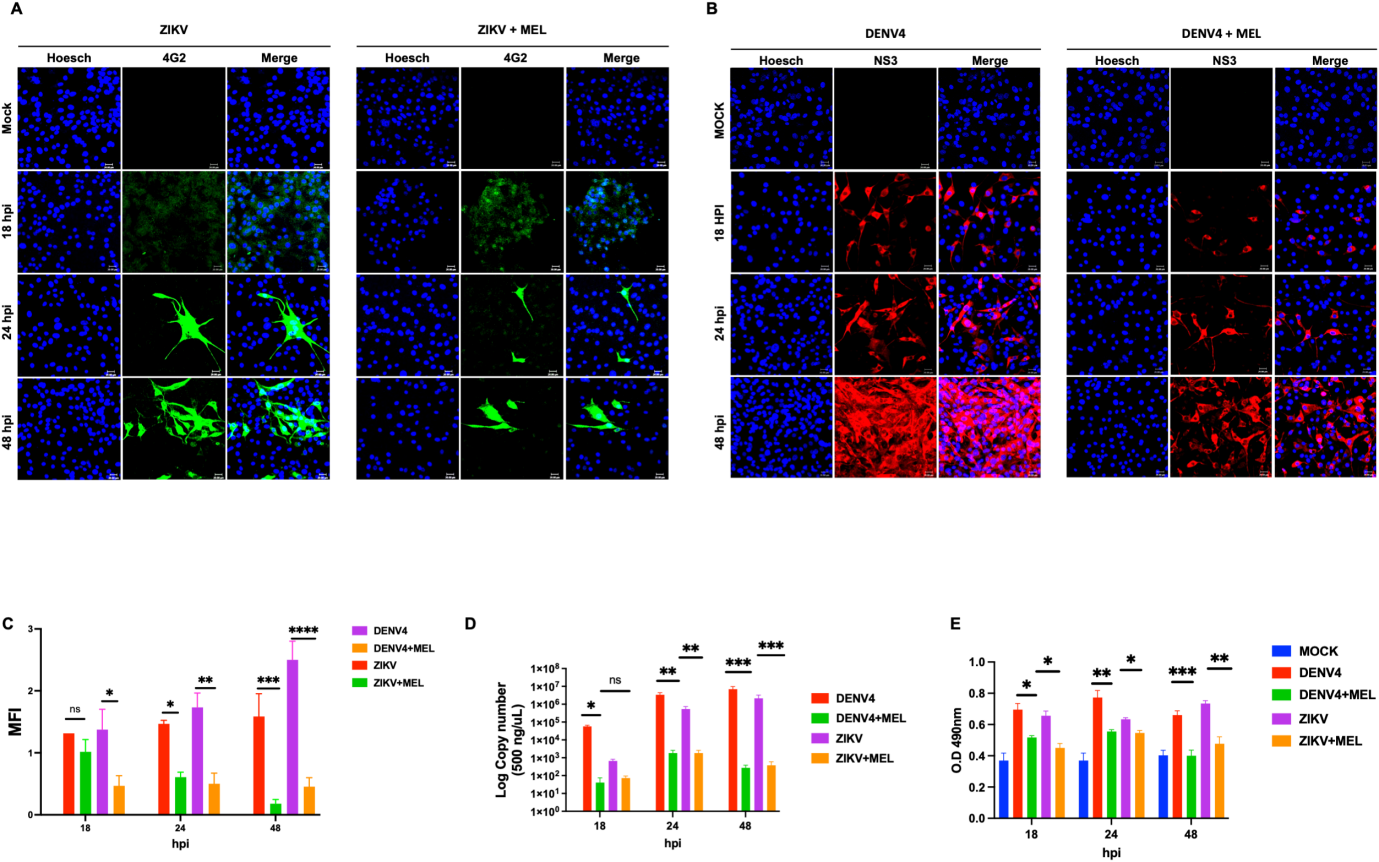
Melatonin 1.2 mM reduces RhoA activation and consequently decreases ZIKV and DENV-4 replication. **(A, B)** Confocal microscopy of U87-MG cells infected with ZIKV (green) and DENV-4 (red) shows a dose-dependent reduction in viral protein expression following melatonin treatment. the mean fluorescence intensity **(C)**. Nuclei were counterstained with Hoechst (blue), compared to untreated infected controls. These findings were supported by qPCR analysis of viral genome copies **(D)** and ELISA-based quantification of RhoA activation **(E)**. Bar graphs represent mean values ± SD from three independent replicates. Asterisks indicate statistical significance of Mann–Whitney test: ns = not significant, *p ≤ 0.05, **p ≤ 0.01, ***p ≤ 0.001, ****p ≤ 0.0001.

Considering that both ZIKV and DENV-4 manipulate cytoskeletal dynamics during infection, we evaluated whether MEL modulates RhoA activation. Confocal microscopy revealed that a 1.2 mM dose effectively reduced viral protein expression (ZIKV envelope and DENV-4 NS3) (**Figures 4A, B**), with a corresponding decrease in MFI at 48 hpi (**Figure 4C**). qPCR analysis corroborated a sustained decrease in viral genome copies over time (18, 24, and 48 hpi) (**Figure 4D**).

Kinetic ELISA analysis demonstrated significant activation of RhoA upon ZIKV and DENV-4 infection, which was significantly reduced by melatonin treatment at 24 and 48 hpi (**Figure 4E**). These findings suggest that melatonin inhibits viral replication at least partly through suppression of RhoA signaling.

### 3.5 Melatonin prolonged the survival of immunodeficient AG129 mice during ZIKV and DENV-4 infections

**Figure 5:**
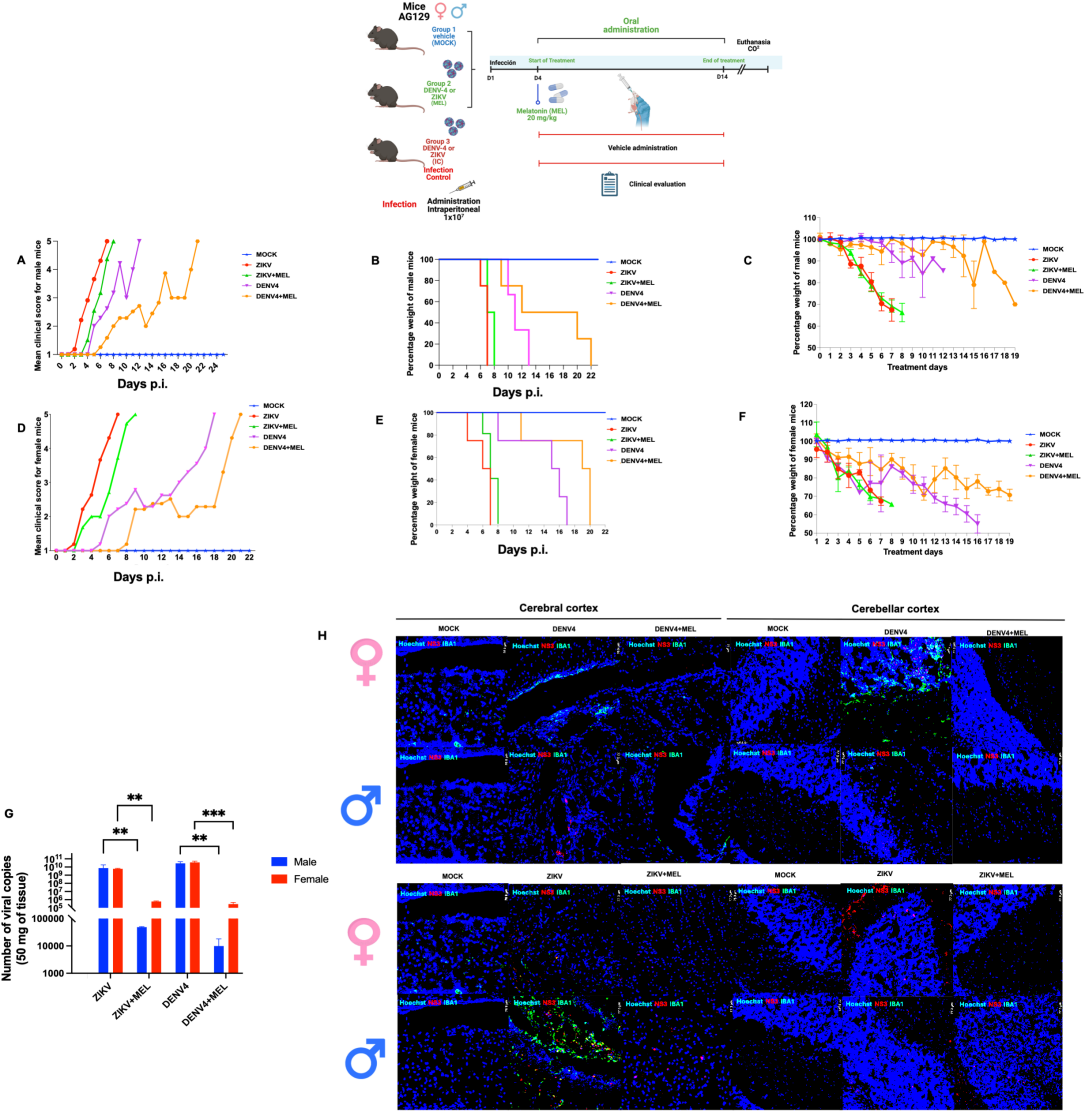
Effect of melatonin (MEL, 20 mg/kg) treatment on AG129 mice infected with ZIKV or DENV-4 survival. The “MOCK” group consisted of uninfected mice treated with MEL; the “ZIKV” or “DENV-4” group included infected mice receiving water (control); and the “ZIKV + MEL” or “DENV-4 + MEL” group included infected mice treated with MEL. **(A, D)** Kaplan–Meier survival curves; **(B, E)** mean clinical scores; and **(C, F)** average body weight percentages in AG129 mice infected with ZIKV **(A–C)** or DENV-4 **(D–F)**. **(G)** Viral load in the brains of infected mice was quantified by qRT-PCR using total RNA. **(H)** Fluorescent immunohistochemistry (IHC) of female and male mice infected with DENV-4. Bar graphs represent mean values ± SD from three independent replicates. Asterisks indicate statistical significance of Mann–Whitney test: ns = not significant, *p ≤ 0.05, **p ≤ 0.01, ***p ≤ 0.001, ****p ≤ 0.0001.

To evaluate the protective effect of MEL in an *in vivo* model, AG129 mice were infected with ZIKV or DENV-4 and treated with 20 mg/kg MEL. In ZIKV-infected mice (**Figures 5A–F**), mild symptoms appeared early, progressing rapidly to severe neurological impairment and death. Although no significant differences in survival were detected (**Table 2**), melatonin-treated mice exhibited delayed symptom onset and slightly. In the DENV4 model, MEL treatment significantly increased the mean survival from 12.25 to 16.25 dpi and Along with delayed clinical signs and maintained body weight and lower viral genome copies in the brain. In the ZIKV model, although survival differences were not statistically significant, MEL-treated mice showed a slight increase in average survival 7.6 to 6.75 dpi (**Table 2**), along with delayed clinical signs, and to body weight maintenance (**Figures 5B, C**). Moreover, quantitative analysis revealed lower viral loads of ZIKV and DENV-4 in the brains of animals treated with melatonin.

Table 2Summary of statistical test results for the experimental groups: ZIKV (n=8), ZIKV+MEL (n=8), DENV-4 (n=8), and DENV-4+MEL (n=8).

**Table:** 

Treatment	MOCK		ZIKV		ZIKV+MEL		DENV-4		DNV-4+MEL	
Sex	M	F	M	F	M	F	M	F	M	F
n	4	4	4	4	4	4	4	4	4	4
Total	8		8		8		8		8	
Median survival (days)	25	25	7	7	7.5	7.5	10	15.5	16	18.5
Total	25		7		7.5		12.76		17.25	
Average survival rate (days)	25	25	6.75	6.75	7.5	7.5	10.5	14.0	15.75	16.75
Total	25		6.75		7.5		12.25		16.25	

Male group	Mantel-Cox	p value	significance
ZIKV *vs* ZIKV+MEL	1.60	0.20	ns
DENV4 *vs* DENV4+MEL	5.58	0.01	*

Male group	ANOVA-LSD	p value	significance
ZIKV *vs* ZIKV+MEL	0.45	0.51	ns
DENV4 *vs* DENV4+MEL	6.23	0.02	*

Female group	Mantel-Cox	p value	significance
ZIKV *vs* ZIKV+MEL	1.90	0.16	ns
DENV4 *vs* DENV4+MEL	7.15	0.007	*

Female group	ANOVA-LSD	p value	significance
ZIKV *vs* ZIKV+MEL	0.72	0.41	ns
DENV4 *vs* DENV4+MEL	8.45	0.007	*

*p < 0.05, **p < 0.01, ***p < 0.001.

Groups were compared using Mantel-Cox and ANOVA-LSD tests to assess differences in survival. The MOCK+MEL was included as a negative control to evaluate the toxicity of melatonin treatment in non-infected animals.

In DENV-4 infected mice (**Figures 5A–F**), melatonin significantly improved survival rate (16.25 *vs*. 12.25 days, treated and untreated, respectively; **Table 2**), delayed symptom onset, preserved body weight, and reduced brain viral loads (**Figure 5G**). Fluorescent immunohistochemistry (IHC) of brain and cerebellar tissues from DENV-4-infected male and female mice revealed greater IBA1 activation in females compared to males, correlating with observed survival outcomes (**Figure 5H**). MEL-treated mice exhibited decreased IBA1 and NS3 signals, suggesting reduced neuroinflammation and viral replication. The slower progression of disease in DENV-4 infection may have contributed to the enhanced therapeutic efficacy of melatonin. In ZIKV-infected mice (**Figure 5H**), IBA1 expression was higher in males compared to melatonin-treated counterparts, while females displayed stronger NS3 signals, indicative of higher viral burden. Importantly, both male and female mice treated with melatonin showed reduced IBA1 and NS3 expression, supporting its potential role in limiting neuroinflammation and viral replication in the brain and cerebellum.

### 3.6 Melatonin reduces neuroinflammation and viral replication in neonatal immunocompetent CD1 mice

**Figure 6:**
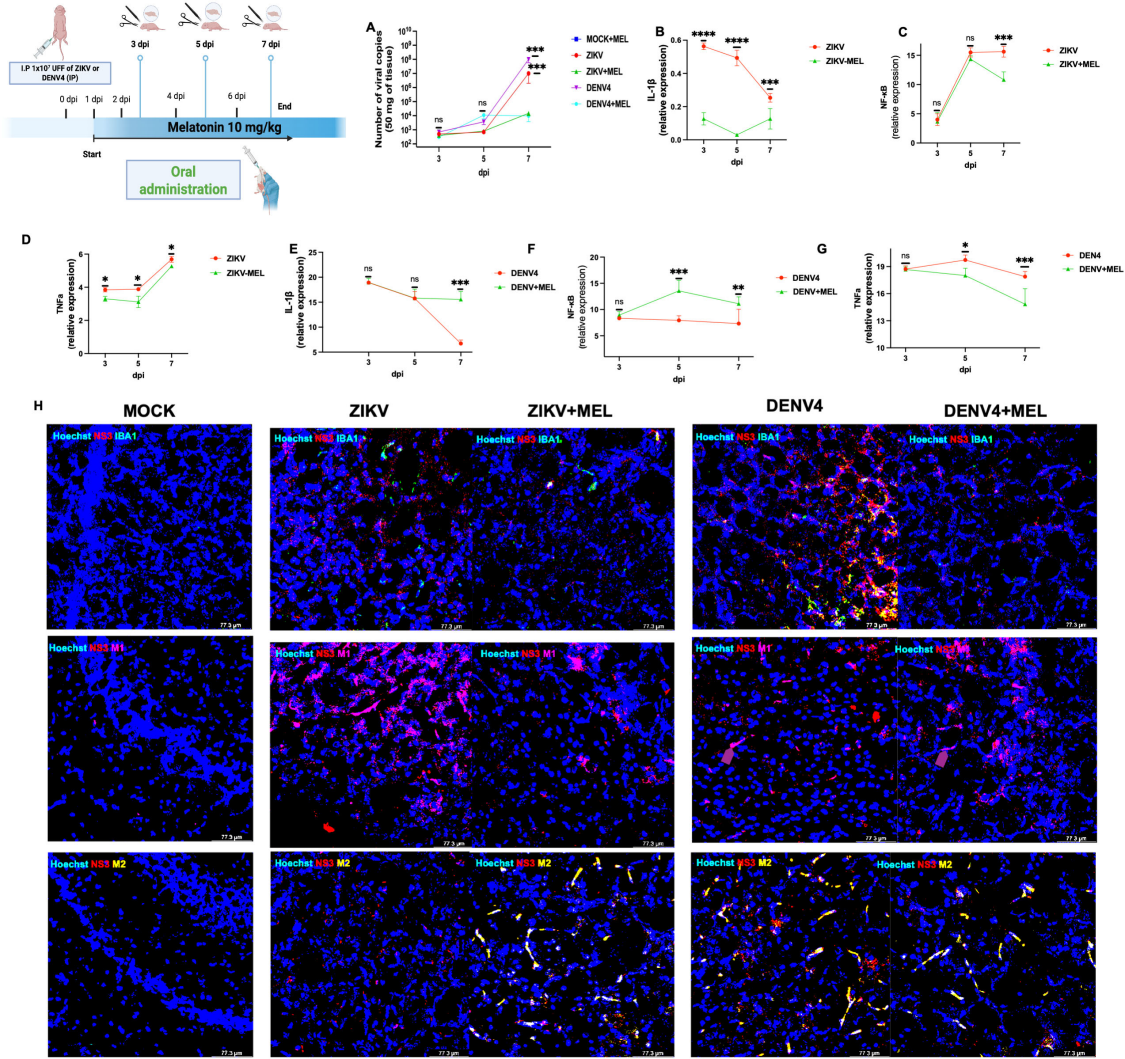
Neuroprotective and neuroimmunomodulator effects of melatonin (10 MEL, mg/kg) in immunocompetent neonatal CD1 mice. Neonatal CD1 mice were processed in groups of three per condition. Total RNA was extracted from the left-brain lobe of each animal, and qPCR was performed to determine viral genome copies using a logarithmic standard curve **(A)**. In addition, pro-inflammatory immune response panels were evaluated in brain tissue from mice infected with ZIKV **(B–D)**, DENV-4 **(E–G)** and differentially modulates microglial M1/M2 polarization in cerebral cortex at 7 dpi **(H)**. Bar graphs represent mean values ± SD from three independent replicates. Asterisks indicate statistical significance of Mann–Whitney test: ns, not significant, *p ≤ 0.05, **p ≤ 0.01, ***p ≤ 0.001, ****p ≤ 0.0001.

Finally, we tested melatonin in a transient infection model using neonatal immunocompetent CD1 mice. Following intraperitoneal infection with ZIKV or DENV-4, viral RNA was detectable at 3 dpi and peaked at 7 dpi (10^7^ copies for ZIKV; 10^8^ for DENV-4) (**Figure 6A**). MEL treatment (10 mg/kg) substantially reduced viral loads by 3–4 log units (**Figure 6A**).

Expression analysis of neuroinflammatory markers revealed that melatonin reduced IL-1β, TNFα, and NF-κB during ZIKV infection (**Figures 6B–D**). During DENV-4 infection, melatonin reduced TNFα but increased IL-1β and NF-κB expression at 7 dpi (**Figures 6E–G**). These findings suggest that MEL can modulate neuroinflammation and inhibit viral replication in the cerebral cortex, supporting its therapeutic potential against Orthoflavivirus induced neuroinfections.

### 3.7 Melatonin differentially modulates microglial M1/M2 polarization in ZIKV and DENV-4 neuroinfections at cerebral cortex of neonatal immunocompetent CD1 mice

Several studies have shown that the RhoA signaling pathway is involved in regulating M1 and M2 polarization of macrophages and microglia (19–21). Although the precise mechanisms underlying this phenotypic switch and cellular activation remain debated, few investigations have directly addressed the role of RhoA as a neuroprotective factor during ZIKV and DENV-4 neuroinfections.

To further explore this, we assessed proinflammatory and anti-inflammatory markers in cerebral cortex of neonatal immunocompetent CD1 mice at 7 dpi. IBA1 (green) and CD86 (purple) were used to identify the proinflammatory M1 microglial phenotype, while CD206 (yellow) served as a marker of the anti-inflammatory M2 phenotype. Our results revealed distinct neuroimmune responses between ZIKV and DENV-4 infections. ZIKV infection was associated with increased expression of IBA1 and CD86, indicative of predominant M1 microglial activation. However, melatonin treatment shifted this profile toward an M2 phenotype, as evidenced by elevated CD206 expression, suggesting that melatonin promotes inflammation resolution and tissue repair during ZIKV infection (**Figure 6H**, right).

In contrast, DENV-4 infection induced a mixed microglial response, characterized by increased IBA1 and CD206 expression and low CD86 levels. Notably, melatonin treatment in DENV-4 infected mice resulted in decreased IBA1 and CD206 expression, alongside increased CD86 levels, indicating a shift toward a proinflammatory M1 phenotype (**Figure 6H**, left). These findings support the notion that melatonin exerts differential immunomodulatory effects in the brain, promoting an M2 phenotype in ZIKV infection and an M1 phenotype in DENV-4 infection. This differential modulation aligns with our qPCR data, which showed increased expression of proinflammatory genes such as IL-1β and NF-κB following melatonin treatment (**Figures 6B–G**).

## 4 Discussion

Currently, no effective vaccine or safe specific antiviral treatment exists for ZIKV or DENV-4 infections. Moreover, available therapeutic strategies do not specifically address the neurological manifestations associated with these viruses. In this context, drug repurposing strategies using FDA-approved compounds represent a promising therapeutic avenue, offering advantages such as reduced development time and well-established safety profiles.

Among potential targets, the RhoA pathway has been extensively studied in the nervous system (7, 19), where it regulates cytoskeletal dynamics and immune responses. MEL has been shown to negatively modulate RhoA activation in several neuroinflammatory conditions, leading to improved clinical outcomes (20–22). Our findings demonstrate that ZIKV and DENV-4 infections significantly activate RhoA signaling in U87-MG cells, suggesting that RhoA plays a critical role during infection, particularly in viral replication and the maturation of infectious particles. The difference between viral genome copy numbers and focus-forming units (FFUs) may be attributed to differences in sample processing. For genome quantification, the entire cell monolayer is harvested, capturing both mature and immature viral particles within the cells. In contrast, the FFUs assay analyzes the cell culture supernatant, which predominantly contains mature viral particles, typically representing only about 10% of the total viral particles present in the infected cells. This discrepancy likely explains the lower FFU counts relative to genome copy numbers (1).

Consistent with these observations, treatment with the RhoA-specific inhibitor CT04 markedly reduced viral protein expression, infectious particle production, and viral genome copy numbers. These results align with previous studies involving the JEV, where RhoA inhibition similarly reduced viral entry and replication (9). Importantly, while prior studies applied RhoA inhibitors before infection, our model treated cells post-infection (at 48 hpi), yet still observed significant antiviral effects, reinforcing the relevance of RhoA as a conserved target across Orthoflavivirus.

Among compounds capable of modulating RhoA, melatonin stands out due to its neuroprotective, antioxidant, and immunomodulatory properties (24). Previous studies have described melatonin’s antiviral activity against viruses such as Ebola (26), SARS-CoV-2 (25), and different dengue serotypes (27), including ZIKV (28). However, its effects in an *in vivo* model during Orthoflavivirus neuroinfections had not been fully demonstrated.

*In vitro*, we found that MEL exhibited clear antiviral effects at both 0.6 and 1.2 mM concentrations, with 1.2 mM being the most effective. Although typical therapeutic doses in humans do not reach 1.2 mM, our results suggest that melatonin may have antiviral effects beyond its circadian role, especially under conditions where local tissue concentrations are elevated. This antiviral action was reflected by reduced viral protein levels, lower infectious titers, and decreased viral RNA levels. These findings are consistent with previous reports showing that melatonin treatments reduced ZIKV replication in neuron-derived cells at similar concentrations (28) and limited DENV-4 particle release (27), suggesting a conserved antiviral mechanism.

Importantly, our study extended beyond antiviral effects, exploring the immunomodulatory actions of MEL during infection. MEL altered the expression of pro-inflammatory genes and interferon-stimulated genes (ISGs) in U87-MG cells. During ZIKV infection, MEL reduced TNFα, MX1, and IFI44L levels. We observed a relative increase in the expression of IL-1β and IFNβ. This result may be explained by the complex regulatory relationship between these two cytokines, as previous studies have reported that IL-1β expression can be downregulated by IFNβ. Thus, the simultaneous increase of both cytokines might reflect a specific phase of their regulatory interplay during infection (28–30) Although we are assessing gene expression rather than protein levels, this mechanism may still suggest such regulatory dynamics. In DENV-4 infection, MEL consistently down-regulated all evaluated pro-inflammatory and ISG transcripts, highlighting virus-specific modulation.

Although the ELISA kit used does not include a standard calibration curve, the absorbance values (OD) provide a relative measure of endogenous RhoA activation levels. The principle of this assay relies on the ability of RhoA to cycle between an inactive GDP-bound form and an active GTP-bound form. Specifically, the ELISA employs a capture strategy using a Rhotekin-RBD domain immobilized on plates, which selectively binds to active RhoA-GTP. Unbound proteins are washed away, and active RhoA is detected using a specific primary antibody, followed by an HRP-conjugated secondary antibody for colorimetric detection. This approach enables the quantification of relative RhoA activity across various experimental conditions. Consistent with these findings, kinetic analyses of RhoA activation revealed a progressive increase during ZIKV and DENV-4 infections, which was significantly attenuated by MEL treatment from 18 hpi onward. This decrease in RhoA activity correlated with reductions in viral proteins and genome copies, suggesting that MEL impairs viral replication, at least in part, by suppressing RhoA signaling.

The relevance of these findings was tested in AG129 mice, a highly susceptible model due to their lack of IFN-α/β and IFN-γ receptors (13, 31–33). In mice infected with the Zika virus, treatment with MEL delayed the onset of symptoms, preserved body weight, and slightly improved survival; however, statistical significance was not reached, likely due to the small sample size and biological variability. While MEL reduced viral load as well as IBA1 and NS3 expression in the Central Nervous System (CNS) tissues, the therapeutic effect could be enhanced with early or prolonged administration. This is especially relevant given the aggressive neurotropism and rapid disease progression associated with ZIKV infection. Early neuroinvasion can disrupt critical physiological functions, such as food and water intake, thereby intensifying disease severity and reducing the window for effective therapeutic intervention (13, 31, 32).

In contrast, during DENV-4 infection, melatonin (MEL) significantly prolonged survival by approximately four days, delayed the onset of clinical signs, reduced weight loss, and decreased viral load in the brain. The concurrent reduction in IBA1 and NS3 signals in CNS tissues further supports its activity within the central nervous system. These findings suggest that the slower progression of DENV-4 disease allowed MEL to exert more effective antiviral and immunomodulatory effects. Our results revealed a difference in the efficacy of melatonin treatment between female and male mice infected with DENV-4, with a more pronounced antiviral effect observed in males. Metabolic differences could explain this phenomenon, as females generally exhibit higher lipid production and more complex hormonal regulation than males. Previous studies have shown that flaviviruses rely on lipids, such as cholesterol, to support viral replication. Therefore, the increased lipid availability in females may contribute to enhanced viral replication and greater pathogenicity (3).

Our results align with previous studies demonstrating that targeting the RhoA pathway can reduce vascular permeability and improve outcomes during severe dengue (36), supporting the therapeutic relevance of modulating this pathway. MEL, as a safe, FDA-approved molecule, offers an attractive candidate for this approach.

We further validated our findings in a neonatal immunocompetent CD1 mouse model, where ZIKV and DENV-4 established transient brain infections without causing overt disease or mortality (34, 35). MEL treatment reduced viral brain loads by 3–4 log units and modulated neuroinflammatory markers, notably reducing IL-1β, TNFα, and NF-κB during ZIKV infection, and modulating TNFα, IL-1β, and NF-κB during DENV-4 infection.

Although clinical signs were absent in this model, the immunomodulatory effects observed are highly relevant. Immunocompetent models better mimic human infection dynamics, where ZIKV and DENV-4 are generally self-limiting, and severe neurological complications involve disregulated immune responses. Neonatal CD1 mice thus represent a valuable tool for studying host–pathogen interactions and therapeutic modulation (35–37).

The evaluation of M1 and M2 markers in the brains of immunocompetent mice, as well as the use of pharmacological agents such as MEL, remains limited. Nevertheless, our results support the concept of virus-specific immune modulation induced by ZIKV and DENV-4. While DENV-4 strongly induces a mixed M1/M2 microglial response, ZIKV infection predominantly promotes M1 polarization. Our data demonstrate that MEL attenuates proinflammatory pathways: during ZIKV infection, it promotes M2 polarization, whereas in DENV-4 infection, it reduces IBA1 activation. However, the precise mechanisms underlying the M1/M2 phenotypic switch remain poorly understood.

These observations align with previous reports showing that both viruses drive M1 polarization in severe cases of ZIKV and DENV associated encephalitis, and that inhibition of specific signaling pathways can alter this balance (38). Moreover, in models of neurodegenerative diseases, RhoA inhibition has been associated with reduced M1 activation, facilitating inflammation resolution, and limiting tissue damage (39). Taken together, these findings suggest that RhoA and other inflammatory mediators may represent key therapeutic targets for controlling neuroinflammation during Orthoflavivirus infections. In this context, MEL emerges as a promising pharmacological candidate capable of modulating the RhoA pathway.

The use of a commercial liquid melatonin preparation like the one we used in this study guarantees the antiviral and immunomodulatory effect of commercial melatonin presentations that are available without a prescription and are inexpensive. Based on this calculation, the 10–20 mg/kg doses in mice correspond to approximately 0.8–1.6 mg/kg in humans, or 56–112 mg for a 70-kg adult. These values fall within the range of high-dose melatonin regimens used in clinical studies, where doses up to 1000 mg/day have been administered safely and without major toxicity (40).

Collectively, our results highlight the central role of host signaling pathways, particularly RhoA, in regulating ZIKV and DENV-4 replication. They support the repurposing of MEL as a dual antiviral and immunomodulatory agent. Furthermore, they suggest that targeting host factors like RhoA may offer a broad-spectrum (pan-flavividae) therapeutic strategy (41–44).

Future studies should focus on elucidating the precise molecular mechanisms by which RhoA supports Orthoflavivirus replication, testing MEL efficacy in other models of neuroinfection and exploring combination therapies that target both viral and host factors.

Our findings provide a foundation for developing safe, effective therapeutic interventions for Orthoflavivirus neuroinfections, leveraging the modulation of host signaling pathways.

## Data Availability

The raw data supporting the conclusions of this article will be made available by the authors, without undue reservation.

## Supplementary material

Supplementary Figure 1 Evaluation of clinical signs of ZIKA and DENV-4 infection in AG129 mice. Clinical progression was monitored daily in AG129 mice following infection with ZIKV (1 ×10^6^ FFU) or DENV-4 (1 ×10^7^ FFU), beginning on day four post-inoculation. Characteristic signs of disease—including ruffled fur, hunched posture, reduced mobility, limb weakness, and weight loss—were evaluated. The severity of clinical signs was scored using a standardized scale, and cumulative scores were calculated. This assessment enabled the comparison of disease progression and symptom severity between ZIKV and DENV-4 infections.

Supplementary Figure 2 Cytotoxicity (CC_50_) of CT04 Inhibitor in U87-MG cells. U87-MG cells were seeded in 96-well plates at 70% confluence and treated for 48 hours with vehicle control or CT04 Inhibitor at concentrations of 1 and 2 μg/mL. Cell viability remained above 80% at all concentrations tested. Cytotoxicity was assessed using the MTT assay and quantified by absorbance measurements. Bar graphs represent mean values ± SD from three independent replicates. Asterisks indicate statistical significance of Mann–Whitney test: ns = not significant, *p ≤ 0.05, **p ≤ 0.01, ***p ≤ 0.001, ****p ≤ 0.0001.

Supplementary Figure 3 Cytotoxicity (CC_50_) of melatonin in U87-MG cells. U87-MG cells were seeded in 96-well plates at 70% confluence and treated for 48 hours with vehicle control or melatonin (MEL) at concentrations of 0.15, 0.3, 0.6, and 1.2 mM. Cell viability remained above 80% at all concentrations tested. Cytotoxicity was assessed using the MTT assay and quantified by absorbance measurements. Bar graphs represent mean values ± SD from three independent replicates. Asterisks indicate statistical significance of Mann–Whitney test: ns = not significant, *p ≤ 0.05, **p ≤ 0.01, ***p ≤ 0.001, ****p ≤ 0. 0001.
