# Supplementary figures and images for: Pharmacological inhibition of the RhoA pathway by melatonin reduces viral replication and proinflammatory response against ZIKV and DENV-4 neuroinfections
Source: Front Immunol. 2025 Aug 1;16:1630116. doi: 10.3389/fimmu.2025.1630116 (PMC12354632; doi:10.3389/fimmu.2025.1630116)

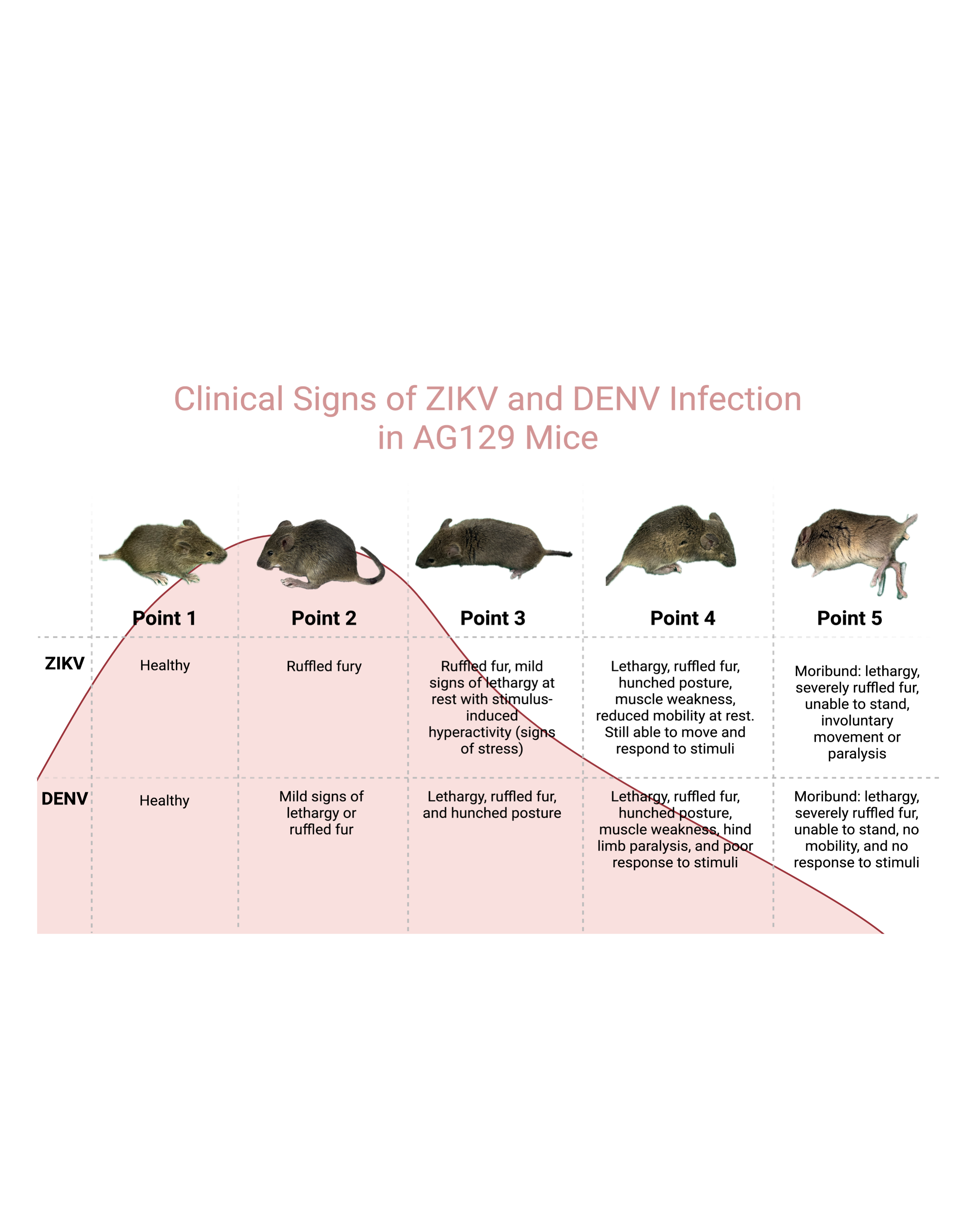

Supplement: Supplementary Figure 1 — Evaluation of clinical signs of ZIKA and DENV-4 infection in AG129 mice. Clinical progression was monitored daily in AG129 mice following infection with ZIKV (1 ×10^6^ FFU) or DENV-4 (1 ×10^7^ FFU), beginning on day four post-inoculation. Characteristic signs of disease—including ruffled fur, hunched posture, reduced mobility, limb weakness, and weight loss—were evaluated. The severity of clinical signs was scored using a standardized scale, and cumulative scores were calculated. This assessment enabled the comparison of disease progression and symptom severity between ZIKV and DENV-4 infections. [file Image1.tiff]

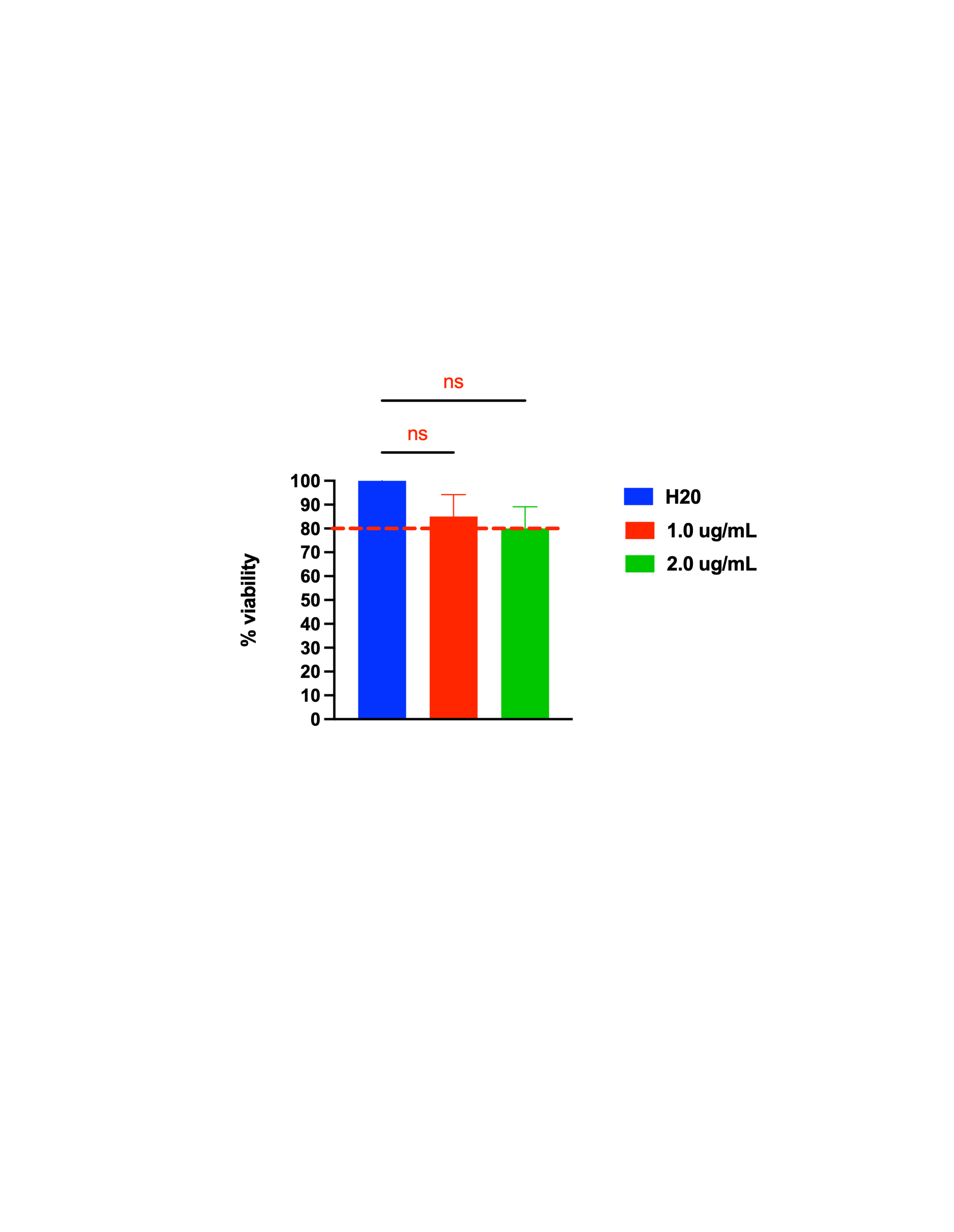

Supplement: Supplementary Figure 2 — Cytotoxicity (CC50) of CT04 Inhibitor in U87-MG cells. U87-MG cells were seeded in 96-well plates at 70% confluence and treated for 48 hours with vehicle control or CT04 Inhibitor at concentrations of 1 and 2 μg/mL. Cell viability remained above 80% at all concentrations tested. Cytotoxicity was assessed using the MTT assay and quantified by absorbance measurements. Bar graphs represent mean values ± SD from three independent replicates. Asterisks indicate statistical significance of Mann–Whitney test: ns = not significant, *p ≤ 0.05, **p ≤ 0.01, ***p ≤ 0.001, ****p ≤ 0.0001. [file Image2.tiff]

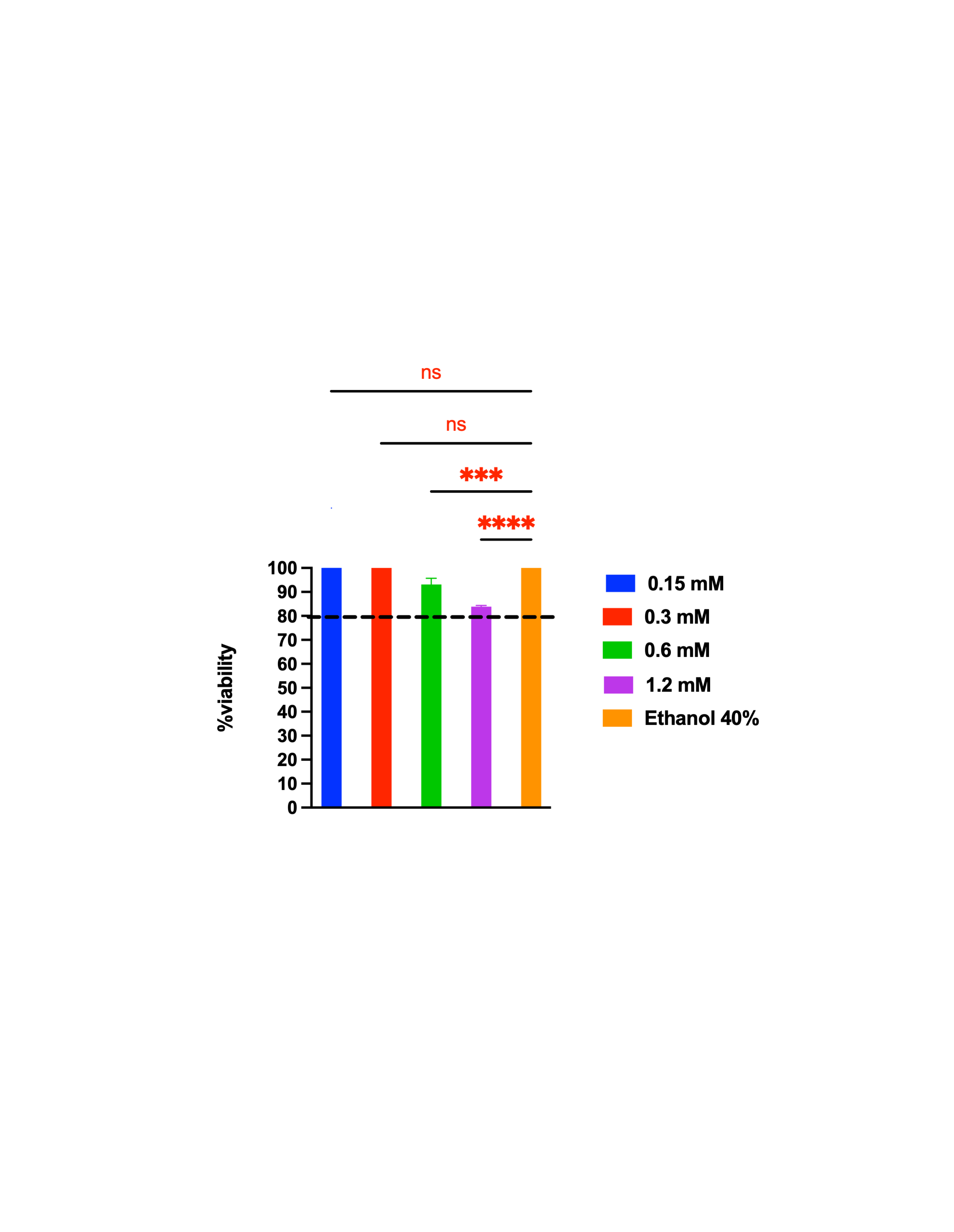

Supplement: Supplementary Figure 3 — Cytotoxicity (CC50) of melatonin in U87-MG cells. U87-MG cells were seeded in 96-well plates at 70% confluence and treated for 48 hours with vehicle control or melatonin (MEL) at concentrations of 0.15, 0.3, 0.6, and 1.2 mM. Cell viability remained above 80% at all concentrations tested. Cytotoxicity was assessed using the MTT assay and quantified by absorbance measurements. Bar graphs represent mean values ± SD from three independent replicates. Asterisks indicate statistical significance of Mann–Whitney test: ns = not significant, *p ≤ 0.05, **p ≤ 0.01, ***p ≤ 0.001, ****p ≤ 0. 0001. [file Image3.tiff]
